# Context, Content, and the Occasional Costs of Implicature Computation

**DOI:** 10.3389/fpsyg.2019.02214

**Published:** 2019-10-25

**Authors:** Raj Singh

**Affiliations:** Institute of Cognitive Science, Carleton University, Ottawa, ON, Canada

**Keywords:** implicature, exhaustivity, complexity, processing, questions and answers, ambiguity

## Abstract

The computation of scalar implicatures is sometimes costly relative to basic meanings. Among the costly computations are those that involve strengthening “some” to “not all” and strengthening inclusive disjunction to exclusive disjunction. The opposite is true for some other cases of strengthening, where the strengthened meaning is *less* costly than its corresponding basic meaning. These include conjunctive strengthenings of disjunctive sentences (e.g., free-choice inferences) and exactly-readings of numerals. Assuming that these are indeed all instances of strengthening via implicature/exhaustification, the puzzle is to explain why strengthening sometimes increases costs while at other times it decreases costs. I develop a theory of processing costs that makes no reference to the strengthening mechanism or to other aspects of the derivation of the sentence's form/meaning. Instead, costs are determined by domain-general considerations of the grammar's output, and in particular by aspects of the *meanings* of ambiguous sentences and particular ways they update the context. Specifically, I propose that when the hearer has to disambiguate between a sentence's basic and strengthened meaning, the processing cost of any particular choice is a function of (i) a measure of the semantic complexity of the chosen meaning and (ii) a measure of how much relevant uncertainty it leaves behind in the context. I measure semantic complexity with Boolean Complexity in the propositional case and with semantic automata in the quantificational case, both of which give a domain-general measure of the minimal representational complexity needed to express the given meaning. I measure relevant uncertainty with the information-theoretic notion of entropy; this domain-general measure formalizes how ‘far' the meaning is from giving a complete answer to the question under discussion, and hence gives an indication of how much representational complexity is yet to come. Processing costs thus follow from domain-general considerations of current and anticipated representational complexity. The results might also speak to functional motivations for having strengthening mechanisms in the first place. Specifically, exhaustification allows language users to use simpler forms than would be available without it to both resolve relevant uncertainties and convey complex meanings.

## 1. Introduction

### 1.1. Basic and Strengthened Meanings

It is commonly assumed that the ‘basic meaning' of the sentence in (1)—the meaning as compositionally derived using the lexical items overtly present in the sentence—is the existential meaning ∃ in (1-a) that we learn in introductory logic. The sentence can of course be used to convey that Jan did not eat all of the cookies, ¬∀. This is not entailed by the sentence's basic meaning. Instead, the inference is commonly assumed to be an inference called the ‘scalar implicature' of ∃ (1-b). Scalar implicatures are computed by a general mechanism that reasons about alternative propositions the speaker could have expressed but chose not to (in this case that Jan ate all of the cookies). The conjunction of (1)'s basic meaning with its scalar implicature is its “strengthened meaning” (1-c).

(1) Jan ate some of the cookies
  - a. Basic meaning: that Jan ate some, possibly all, of the cookies (= ∃)
  - b. Scalar implicature: that Jan did not eat all of the cookies (= ¬∀)
  - c. Strengthened meaning: that Jan ate some but not all of the cookies (= ∃ ∧ ¬∀).

There is debate about the mechanism responsible for strengthening. For example, there are questions about whether the mechanism is part of the linguistic system itself or is shorthand for pragmatic or central-system reasoning. Putting this architectural question aside for the moment, all agree that the mechanism is an alternative-sensitive computation. More precisely, it is commonly assumed that there is a function, *STR*, which computes strengthened meanings by conjoining the sentence *S* with the negation of some of the alternatives of *S*, *ALT*(*S*)^1^. In general, *STR* is thought to be sensitive to various contextual factors, such as what is relevant, what is salient, what is assumed about the speaker's epistemic state, and other factors that have been identified in the literature. Thus, *STR* is a function that takes at least three inputs: the sentence *S*, its alternatives *ALT*(*S*), and the context *c*, and returns the strengthened meaning of *S* in *c*, $S_{c}^{+}$: $STR\left(S,ALT\left(S\right),c\right)=S_{c}^{+}$. Thus, in a context *c* in which ∀ is relevant and the speaker is assumed to be opinionated about whether ∀ is true, $STR\left(\exists ,ALT\left(\exists \right),c\right)=\exists_{c}^{+}=\exists \wedge \neg \forall$. Suppose, however, that ∃ is uttered in a context *c*′ in which ∀ isn't even relevant. In such a case, we say that the context has “pruned” ∀ from *ALT*(∃) (for more on pruning and constraints on pruning, see e.g., Magri, 2009; Fox and Katzir, 2011; Katzir, 2014; Crnič et al., 2015; Singh et al., 2016b). This pruning means that there are no alternatives left in *ALT* to negate, and hence application of *STR* would have no effect: $STR\left(\exists ,ALT\left(\exists \right),c^{\prime }\right)=\exists_{c^{\prime }}^{+}=\exists$. In what follows, unless otherwise noted I will assume that we are in contexts in which all the members of *ALT* are relevant and that the speaker is opinionated about them. I will also sometimes disregard the distinction between a sentence and its denotation when there is little risk of confusion.

Competence theories of implicature computation need to specify *STR* and *ALT* and their interactions with the context such that the right strengthened meaning is derived for any sentence *S* in any context *c*. I will not spend much time discussing competing theories of these components. My concern in this paper is with exploring how competence-theoretic assumptions about strengthening might be realized in performance (see Chomsky, 1965 on the competence-performance distinction, and see Chemla and Singh (2014a,b) for the connection to experimental work on scalar implicature). As we will see, my strategy is to focus on the *output* of strengthening, not on the way in which strengthened meanings are actually derived. Specifically, I will explore the hypothesis that the processing costs that are sometimes associated with strengthening are derived entirely from considerations of the *meaning* of the sentence and specific ways in which it updates the context. The computational history of the sentence and its meaning will be irrelevant.

Nevertheless, to fix ideas it will be useful to assume a particular competence-theoretic framework. I will assume without discussion that *STR* is identified with the covert exhaustive operator *exh* proposed in Fox (2007), and that *ALT* is identified with the tree edit operations outlined in Fox and Katzir (2011). This means that the condition that any element *p*∈*ALT*(*S*) needs to satisfy for it to become an actual implicature is that it needs to be ‘innocently excludable' [as Fox (2007) defines the term; see below for illustrative examples]. This also means that alternatives are derived by substitution operations that replace focused nodes with subconstituents (for non-terminals) and with other lexical items (for terminals). My proposal about processing, however, will be compatible with different theories of *STR* and *ALT*; as noted above, the model I develop is concerned with the *inferences* that are generated, rather than the *mechanisms* that give rise to the inferences. This should make my proposal usable for scholars with other ideas about *STR* and *ALT* and their relation to the context of use.

Returning to (1), the basic/strengthened ambiguity follows from a systematic structural ambiguity: the sentence may or may not be parsed with *exh*. If *exh* is left off the parse, the sentence receives its basic meaning, and if *exh* is merged to the parse, the sentence receives its strengthened meaning. Following Fox (2007), the strengthened meaning of a sentence can often be paraphrased by adding *only* to the sentence (and focusing the relevant scalar item). Thus, *Jan ate only some of the cookies* and the strengthened meaning of (1) both convey (1-c). With both *exh* and *only*, *ALT*(∃) = ∀ [by replacing *some* with *all* in (1)]. The question now is whether ∀ is innocently excludable. To test whether ∀ is innocently excludable, the mechanism negates it and examines whether the result of conjoining it with ∃ is consistent. The proposition ∃ ∧ ¬∀ is consistent, and hence the strengthened meaning ∃ ∧ ¬∀ is derived.

Innocent exclusion in this case was straightforward, but the mechanism is motivated by cases where non-trivial decisions need to be made about which alternatives to negate. Disjunctive sentences provide an illustrative example. Note that since *exh* is general, it can apply to any sentence: *S* → *exh*(*S, ALT*(*S*))^2^. The classic inclusive-exclusive ambiguity in disjunction, then, can be accounted for by the presence or absence of *exh*: without *exh*, the sentence receives the basic inclusive meaning in (2-a), and with *exh* the sentence receives its strengthened exclusive meaning in (2-c) by denying the alternative that Mary ate cake and ice-cream (2-b).

(2) Maria ate cake or ice-cream
  - a. Basic meaning: *p* ∨ *q* (inclusive disjunction)
  - b. Scalar implicature: ¬(*p* ∧ *q*)
  - c. Strengthened meaning: (*p* ∨ *q*) ∧ ¬(*p* ∧ *q*) (i.e., the exclusive disjunction *p* ⊕ *q*)

The set of alternatives for (2) is richer than the set of alternatives for (1). Here, as in (1), we have an alternative derived by lexical substitution: *or* is replaced by *and* to yield the conjunction *p* ∧ *q*. However, unlike (1), we have alternatives derived by replacing the root node by its subconstituents *p* and *q*^3^. Thus, *ALT*(*p* ∨ *q*) = {*p, q, p* ∧ *q*}. The computation of innocent exclusion is more involved than with (1). The goal is to find the maximal subset of *ALT*(*p* ∨ *q*) that could be consistently negated with *p* ∨ *q*. We can't negate the entire set, for that would contradict *p* ∨ *q*. There are two maximal consistent exclusions: (i) {*p, p* ∧ *q*}, and (ii) {*q, p* ∧ *q*}. It would be arbitrary to select one of these maximal consistent exclusions over the other. For example, what would justify the negation of *p* over the negation of *q*? The only proposition that appears to be non-arbitrarily excludable is *p* ∧ *q*. A possibly useful motivation behind this idea is to think of (i) and (ii) as two different “votes” for which propositions to exclude. The alternative *p* ∧ *q* is the only one that every vote agrees on, and for this reason it might be thought to be “innocently” excludable. Thus, *p* ∧ *q* gets negated by *exh*, and the strengthened exclusive disjunction meaning (*p* ∨ *q*) ∧ (¬(*p* ∧ *q*)) is derived.

When the alternatives to disjunctive sentences are *not* closed under conjunction, innocent exclusion can assign a *conjunctive* strengthened meaning to disjunctive sentences^4^. Fox (2007) argues that this is the solution to the “paradox” of free-choice inference (Kamp, 1973). I will return to discussion of free-choice and its relation to innocent exclusion in later sections of the paper. I turn my attention now to relating this set of competence-theoretic ideas to performance models.

### 1.2. Processing Costs

At any given stage of the conversation, participants will have to decide whether to merge *exh* (and hence all of its arguments) to the parse of the uttered sentence^5^. To reduce clutter, I will simply write *exh*(*S*) and omit mention of other arguments that *exh* takes, like *ALT*(*S*) and *c*. The hearer thus faces a disambiguation task: they can either parse the sentence as *S* and add meaning [[*S*]] to context *c*, or they can parse the sentence as *exh*(*S*) and add meaning [[*exh*(*S*)]] to *c*. It is plausible to assume that the choice has performance-theoretic consequences, and in particular that strengthened meanings ought to be costlier to process than corresponding basic meanings. To derive the strengthened meaning of sentence *S*, the processor needs to do all the work needed to compute *S* and its basic meaning [[*S*]], and in addition it needs to create *ALT*(*S*), determine which elements of *ALT*(*S*) are innocently excludable, conjoin these innocently excludable propositions with [[*S*]], and—under the identification of *STR* with *exh*—a more complex structure needs to be produced as well (for metrics, see e.g., Miller and Chomsky, 1963; Frazier, 1985, and many others). It would not be unnatural to expect this extra work to be realized in performance difficulties (see Chemla and Singh, 2014a for detailed discussion). To a significant extent, this expectation is borne out, at least with respect to cases like (1) and (2). For example, compared with their basic meanings, the strengthened meanings in (1) and (2) tend to be delayed in reading times in matrix positions (e.g., Bott and Noveck, 2004; Breheny et al., 2006) and in embedded positions (e.g., Chemla et al., 2016), they are late to develop (e.g., Noveck, 2001), they trigger later target looks in eye-tracking (e.g., Huang and Snedeker, 2009), and they are less frequently computed under time pressure (e.g., Bott and Noveck, 2004), under cognitive load (e.g., De Neys and Schaeken, 2007; Marty et al., 2013), and in embedded positions (e.g., Chemla, 2009b; Crnič et al., 2015).

Suppose that we take the above results to broadly indicate that the parser has a harder time with the form-meaning pair < *exh*(*S*), [[*exh*(*S*)]] > than with the form-meaning pair < *S*, [[*S*]] >. Ideally this would follow from a general parsing theory. For example, we might consider the idea that a form-meaning pair λ_1_ = < *f*_1_, *m*_1_ > is easier to process than a form-meaning pair λ_2_ = < *f*_2_, *m*_2_ > if *f*_1_ is contained in *f*_2_ and the computation of *m*_1_ is an intermediate step in the computation of *m*_2_. The challenge would be to motivate the principle from general performance considerations, perhaps along the lines of the traditional “derivational theory of complexity” (see e.g., Fodor et al., 1974 for classic discussion). The core idea would be that processing costs are a monotonically increasing function of syntactic/semantic computational complexity: if the generation of λ_*i*_ involves a proper subset of the computations needed to generate λ_*j*_, then (*ceteris paribus*) the cost of processing λ_*i*_ will be less than the cost of processing λ_*j*_.

There are reasons to doubt that this monotonicity principle is on the right track. First, it appears committed to the assumption that there is a stage at which the parser has considered < *S*, [[*S*]] > as the analysis of the sentence but not < *exh*(*S*), [[*exh*(*S*)]] >. Although natural, other views are also conceivable. For example, under a serial model of processing a single reading is entertained at any given point in processing; if it is found to be undesirable (for whatever reason) it may be replaced by a different reading generated by the grammar. In the case under consideration here, one would have to assume that *exh* appears late in the parser's structure-building. However, one could just as well begin by trying to parse with *exh* and revising only if necessary. This consideration is perhaps even stronger under the assumption that the human sentence processing mechanism uses a parallel processor. Suppose that the parser builds all (or at least many) of the form-meaning pairs that can be assigned to the sentence in a given context, and then decides (or asks the context to decide) which of these to select. Under such a model, the parser will already have produced both the strengthened and unstrengthened meanings, and it is not clear why the strengthened form-meaning pair should have any greater cost associated with it than the unstrengthened pair^6^. Under either view, we would be left with a stipulated “ordering” of computations in need of justification.

More importantly, there is empirical evidence against the monotonicity principle. First, return to the comparison with *only*. Like with *exh*, merging *only* to sentence *S* adds new syntactic and semantic computations. However, *only(S)* is not hard in the way that *exh*(*S*) is. For example, parsing/interpretation of *exh*(*S*) is slower than *only(S)* (e.g., Bott et al., 2012), memory demands inhibit *exh*(*S*) but not *only(S)* (e.g., Marty and Chemla, 2013), and under certain conditions preschool children can compute *only(S)* even though they cannot compute *exh*(*S*) (e.g., Barner et al., 2011). The sentences *exh*(*S*) and *only(S)* involve very similar syntactic and semantic computations. Nevertheless, *exh*(*S*) appears to be systematically harder than *only(S)*.

Taken together, these considerations suggest that costs arise precisely when a listener *chooses* *exh*(*S*) over *S* during disambiguation. When processing *only some*, you cannot choose to understand the sentence as if *only* were not present. When processing (1), you have the option to understand the sentence with and without *exh*. The choice matters, and it appears that the disambiguation mechanism pays some kind of penalty for having chosen < *exh*(*S*), [[*exh*(*S*)]] > over < *S*, [[*S*]] >. This might be taken as evidence for a restricted version of the monotonicity principle that becomes relevant only when the parser has to choose among competing analyses of the sentence. This would then leave us with the challenge of motivating the monotonicity assumption from general processing considerations. However, we will soon see that even this restricted version faces empirical challenges. In particular, the generalization we started with is incorrect: it is not *in general* true that < *exh*(*S*), [[*exh*(*S*)]] > is harder than < *S*, [[*S*]] >. For some constructions, the opposite is true: < *exh*(*S*), [[*exh*(*S*)]] > is sometimes *less costly* than < *S*, [[*S*]] >.

### 1.3. A Puzzle: Scalar Diversity in Processing

Assume that the basic meaning of numerals is an “at least” reading (3-a), and that the “exactly” reading follows from strengthening [(3-b) and (3-c); see Spector (2013) and references therein for relevant discussion of the basic and strengthened meanings of numerals].

(3) Numerals: Sandy ate three of the cookies
  - a. Basic meaning: that Sandy ate at least three of the cookies
  - b. Scalar implicature: that Sandy did not eat at least four of the cookies
  - c. Strengthened meaning: that Sandy ate at least three of the cookies and did not eat at least four of the cookies, i.e., that Sandy ate exactly three of the cookies.

The pattern is thus like with (1) and (2): there is a basic meaning that gets strengthened by *exh*. However, the similarity does not carry over into processing: the strengthened meaning (3-c) is not costly relative to the basic meaning (3-a) (e.g., Huang and Snedeker, 2009; Marty et al., 2013). In fact, Marty et al. (2013) found that there were *more* exactly-readings of numerals under high memory load than under low memory load. This is the exact opposite of “some-but-not-all” type implicatures, which are reduced under high memory load. Thus, burdens on memory resources have the opposite effect for numerals and scalar items like *some*: strengthened meanings are *increased* with numerals and *decreased* with scalars.

Free-choice inferences are another puzzling case. A sentence like (4) has a so-called free-choice inference that Sandy is allowed to eat cake and is allowed to eat ice-cream—Sandy is free to choose (Kamp, 1973). The free-choice inference ◇*p* ∧ ◇*q* does not follow from the logical form ◇(*p* ∨ *q*) if “ ∨ ” is an inclusive disjunction and “◇” is an existential quantifier over possible worlds. It has been argued—for example, on the basis of its sensitivity to monotonicity—that the free-choice inference is a scalar implicature (e.g., Kratzer and Shimoyama, 2002; Alonso-Ovalle, 2005). Various mechanisms have been proposed for deriving (4-c) as the strengthened meaning of (4) (e.g., Fox, 2007; Chemla, 2009a; Franke, 2011; Bar-Lev and Fox, 2017). I will not discuss these here^7^; what is important is that the free-choice inference follows the pattern in (4), and hence is broadly similar to the patterns in (1), (2), and (3).

(4) Free-choice: Sandy is allowed to eat cake or ice-cream
  - a. Basic meaning: ◇(*p* ∨ *q*)
  - b. Scalar implicature: (◇*p* → ◇*q*) ∧ (◇*q* → ◇*p*)
  - c. Strengthened meaning: ◇*p* ∧ ◇*q*.

It turns out that free-choice inferences do not display the processing costs associated with (1) and (2). For example, they are processed faster than and are preferred to their basic meaning counterparts (e.g., Chemla and Bott, 2014), they are more robust under embedding than (Chemla, 2009b), and they are readily computed by children (Tieu et al., 2016). Furthermore, conjunctive strengthenings of disjunctive sentences more generally display these properties: preschool children (e.g., Singh et al., 2016b; Tieu et al., 2017) and adult speakers of Warlpiri (Bowler, 2014) appear to robustly compute conjunctive strengthenings of disjunction^8^.

Let us use “free-choice” to refer to any conjunctive strengthening of disjunction. The challenge we face now is to explain why exhaustification in free-choice and in numerals has the opposite processing consequences than exhaustification in *some* and *or*. This is yet further evidence for a kind of scalar diversity (van Tiel et al., 2016), which takes seriously the observation that scalar implicatures for different constructions sometimes have different properties. Of interest to us here is that we now have evidence for a peculiar competence-performance mismatch:

(5) Competence-uniformity and performance–induced-diversity (CUPID):
  - a. Competence-uniformity: The *competence* system treats the ambiguities in (1)–(4) in a uniform way, characterized as the optional application of a covert operator *exh* that computes innocent exclusion.
  - b. Performance-induced-diversity: In some cases *exh* speeds up processing ((3), (4)), and in other cases it slows down processing (1), (2).

The challenge is to formulate auxiliary assumptions that relate the output of the competence system with measures of processing difficulty such that CUPID is predicted and things no longer seem peculiar. Clearly, any assumptions committed to scalar uniformity in processing will not work. This rules out the monotonicity assumption we were examining earlier under which < *exh*(*S*), [[*exh*(*S*)]] > is generally harder to process than < *S*, [[*S*]] >. It also rules out principles such as the “strongest meaning hypothesis” [e.g., Chierchia et al., 2012, with roots in Dalrymple et al. (1998)] or “charity” (e.g., Meyer and Sauerland, 2009—see also Chemla and Spector, 2011). The goal of this paper is to meet this challenge.

### 1.4. Accounting for CUPID

Previous attempts at accounting for scalar diversity in processing have invariably made reference to language-internal computations and thus in some sense deny CUPID as a challenge to be solved. For example, some accounts have argued that strengthening has a cost when it requires a *lexical substitution* (as in “some but not all”) but not when it requires only constituent substitutions (as in free-choice; e.g., Chemla and Bott, 2014; van Tiel and Schaeken, 2017). The guiding intuition, as I understand it, is that constituents are more readily accessible (they are already in the workspace), whereas lexical substitutions are more costly because the lexicon is presumably less accessible than material you have already created (you need to go out of the workspace to find a new lexical item). These considerations do not extend in any straightforward way to numerals, since their alternatives are derived neither by sub-constituents nor by lexical replacements (the set of numbers is infinite, and hence the alternatives must be referencing the successor function).

Numerals also seem to pose a challenge for the computation-specific proposal in Bar-Lev and Fox (2017). Specifically, they argue that free-choice and scalar implicatures like “some but not all” are derived by two different strengthening computations: roughly, the one for free-choice asserts the truth of alternatives and is context-independent and the mechanism for scalar implicatures negates alternatives and is context dependent. They argue that this distinction can be used to motivate a difference in processing costs. However, so far as I can tell, numerals are like scalar implicatures in the relevant competence-theoretic respects but they nevertheless pattern with free-choice in processing patterns (see also Note 24).

The model in Singh et al. (2016b) also made reference to language-internal computations but it readily accounts for numerals. Specifically, the model considers sets of form-meaning pairs the grammar assigns to the input sentence, and posits two constraints that interact to resolve the ambiguity: one pertaining to the candidate *meanings* and their relation to context, and the other pertaining to the candidate *forms* and their relative complexity. The syntactic assumptions assume the existence of a covert exhaustive operator that furthermore has a special pressure against it. I will discuss this model in greater detail in section 3.1, where I will modify it in various ways in the development of my proposal.

What the above accounts have in common is that they all relate processing costs in one way or another with the strengthening mechanism itself. Here I will pursue a different strategy. I will assume that CUPID teaches us that the costs of exhaustification are *unrelated* to the derivational history of the form/meaning of the sentence. Suppose that the language faculty delivers propositions (sets of worlds) to context-sensitive external systems of thought and action. By focusing our attention on the content produced by the language faculty—rather than on the mechanisms it uses to compute the given content—we might be in better position to develop closer connections between processing costs and arguably non-linguistic tasks like concept learning, theory selection, and communication viewed as a system of information exchange governed by social norms (see Grice, 1967; Fodor, 1983; Chomsky, 1995 among others for relevant discussion). At the same time, the focus on semantic output and context change could make our parsing assumptions relevant to a broader class of theories of the underlying competence system.

The focus on sentence meanings and their relation to contexts allows us to restate the disambiguation problem facing the listener as follows:

(6) Disambiguation as optimal context update: Suppose sentence *S* is uttered in context *c*, and suppose that the grammar G assigns *k* form-meaning pairs to *S*: G(*S*) = { < *f*_1_, *m*_1_ >, …, < *f*_*k*_, *m*_*k*_ >}. These give rise to a candidate set of output contexts C = {*c*_1_, …, *c*_*k*_}, where *c*_*i*_ = *c*+*m*_*i*_ (context *c* updated by *m*_*i*_). The listener's task is to select the optimal element of C as the output context.

This context-update perspective has been found useful in studies of non-determinism in various domains, including parsing (e.g., Fodor, 1983; Crain and Steedman, 1985) and presupposition accommodation (see especially Beaver, 2001; von Fintel, 2008). I hope that it may shed insights into exhaustification decisions as well. Here, I will not say much about the (presumably decision-theoretic) optimality criterion used by the parser in solving (6). Instead, I will focus on the *costs* the parser faces when it chooses to update *c* with a particular *m*_*i*_. There are two costs that I will consider: (i) the *a priori* complexity of *m*_*i*_ as a standalone object, here measured by semantic complexity (see section 2), and (ii) how well *m*_*i*_ resolves relevant uncertainties in *c*, and hence how much relevant uncertainty it leaves in *c*_*i*_, where I identify relevant uncertainty with a function of the number of cells *m*_*i*_ eliminates from the question-under-discussion in *c* (see section 3). The sum of these costs, I argue, solves the challenge raised by CUPID.

## 2. Semantic Complexity

I will begin by pursuing an idea, to my knowledge first suggested in the context of implicature computation by Bott et al. (2012), that the semantic complexity of different pieces of information might be relevant to how hard they are to process. To make this precise, we need an analytic framework that would make clear predictions about how to order different pieces of information for complexity. It turns out that there are branches of mathematical inquiry examining the semantic complexity of propositional and quantificational meanings. Furthermore, these analytical ideas have found useful application in concept learning, which in turn is arguably similar to theory selection and more generally to the choice of one element over some others. Of particular interest is the argument that the semantic complexity of a concept is a good predictor of how easy or hard it is for participants to acquire it (see especially Feldman, 2000 and subsequent work, such as summarized in Piantadosi et al., 2016). These results might thus provide antecedent motivation for the idea that certain pieces of information are intrinsically harder for humans to process than others, and this might be relevant to ordering the costs associated with exhaustification decisions.

### 2.1. Boolean Complexity and Processing Costs

Boolean functions like disjunction and conjunction map sets of truth-values (elements in {0, 1}^*D*^ for any number *D*) to a truth-value (an element in {0, 1}). For example, if *D* = 2, there are four possible combinations of truth-values: {11, 10, 01, 00}. If *D* = 3, there are eight possible combinations: {111, 110, 101, 100, 011, 010, 001, 000}. More generally, there are 2^*D*^ possible combinations of *D* truth-values. Call this *Boolean* *D*-space. A Boolean function maps Boolean *D*-space into {0, 1}. For example, inclusive disjunction maps any element to 1 so long as the element contains at least one 1^9^.

A *Boolean Concept* is the characteristic set of the corresponding Boolean function. A concept is simply a way of carving a domain of interest into those instances that it is true of and those that it is not. For example, *dog* divides the universe into positive instances (things that are dogs) and negative instances (everything else). Similarly, Boolean concepts in *D*-space divide the 2^*D*^ possible truth-value assignments into those that are mapped to true and those that are mapped to false. For example, in Boolean 2-space the positive instances of inclusive disjunction are {11, 10, 01}. Similarly, exclusive disjunction picks out {10, 01}, and conjunction picks out {11}. These concepts, of course, can be thought of as propositions (sets of worlds). For example, the disjunctive concept *p* ∨ *q* is that set of worlds in which either just *p* is true, just *q* is true, or both *p* and *q* are true. We will go back-and-forth between concept talk and proposition talk.

We are interested in examining the extent to which these semantic notions have some intrinsic complexity. When we think of, say, the truth-table method for depicting Boolean functions, it is not immediately obvious why one table should be more or less complex than another. However, there is a perspective—which has been fruitfully applied to empirical facts concerning concept acquisition (Feldman, 2000)—that associates each Boolean concept with an intrinsic complexity measure. The method relates the complexity of a Boolean concept with the *smallest* Boolean formula that can express the concept using negation, inclusive disjunction, and conjunction as primitive (Feldman, 2000)^10^.

(7) Propositional formula: Consider a set of atomic propositional formulae as given. Then the set of propositional formulae is defined recursively as follows:
  - a. Any atom *p* is a formula.
  - b. If *p* is a formula, so is ¬*p*.
  - c. If *p* and *q* are formulae, so is (*p* ∧ *q*).
  - d. If *p* and *q* are formulae, so is (*p* ∨ *q*).

We will sometimes omit parentheses when there is no risk of ambiguity.

(8) The Boolean Complexity of a concept *C* is the length *n* of the smallest formula *f* that expresses *C*: *n* = *min*{|*f*′|:[[*f*′]] = *C*}.
  - a. |*f*′| is the number of *literals* in formula *f*′.
  - b. A *literal* is any atomic formula *p* or its negation ¬*p*.

(9) Examples:
  - a. |(*p* ∨ *q*)| = 2
  - b. |(*p* ∨ ¬*q*)| = 2
  - c. |(*p* ∨ *q*) ∨ (*p* ∧ *q*)| = 4
  - d. |(*p* ∨ *q*) ∧ ¬(*p* ∧ *q*)| = 4
  - e. |(*p* ∧ ¬*q*) ∨ (¬*p* ∧ *q*)| = 4
  - f. |*p* ∧ *q*| = 2.

Clearly, there are many formulae that can express a particular concept. For example, (9-a) and (9-c) both express an inclusive disjunction. However, (9-c) can be simplified to (9-a) without loss of meaning, and (9-a) is the shortest formula that can express inclusive disjunction in Boolean 2-space. There has been significant interest in finding mechanical methods for simplifying propositional formulae (e.g., Quine, 1952, 1955; McCluskey, 1956 and much other work). We will not discuss these here. For our purposes, what is important is that unlike the inclusive disjunction expressed in (9-c), the exclusive disjunction meanings expressed in (9-d) and (9-e) cannot be further compressed (Feldman, 2000). That is, there is no shorter Boolean formula capable of expressing an exclusive disjunction. In this sense, then, exclusive disjunctions are essentially more complex than inclusive disjunctions. They are also more complex than conjunctions [cf. (9-f)].

These complexity results align with empirical observations about the complexity of concept acquisition (again, see Feldman, 2000 and extensive references therein). Specifically, concepts whose membership is determined by an exclusive disjunction (e.g., “pink or square but not both”) are harder to learn than concepts whose membership is determined by inclusive disjunction (“pink or square, possibly both”) and they are also harder to learn than concepts whose membership is determined by conjunction (“pink and square”). This finding suggests that the human mind struggles with exclusive disjunctions in a way that it doesn't with inclusive disjunctions or conjunctions.

Consider now the exhaustification of an inclusive disjunction in the adult state. This leads to an exclusive disjunction interpretation, which we now have reason to think is inherently more complex than its inclusive disjunction counterpart. One way to make sense of the greater difficulty in processing *exh*(*p* ∨ *q*), then, is that it results in a more complex meaning than *p* ∨ *q*. Specifically, it is plausible to assume that the parser incurs a penalty when it chooses to select a complex meaning even though a simpler one was available:

(10) Boolean Complexity and processing costs during disambiguation: Suppose that the grammar G assigns *k* analyses to sentence *S*: G(*S*) = {λ_1_, …, λ_*k*_}, where each λ_*i*_ is a form-meaning pair < *f*_*i*_, *m*_*i*_ >. Let *B*(*m*) be the Boolean Complexity of meaning *m*. Then the *cost of selecting* λ_*i*_ ∈ G(*S*), *C*(λ_*i*_), is proportional to the Boolean Complexity of meaning *m*_*i*_: *C*(λ_*i*_) ∝ *B*(*m*_*i*_).

Note that the formulation in (10) only predicts processing costs that arise from disambiguation *decisions*. It would apply, then, to saying why *exh*(*p* ∨ *q*) is more costly to process than *p* ∨ *q* when the speaker utters a disjunctive sentence *p or q*, but it would not say anything about the relative cost of processing *only(p or q)* because no disambiguation is involved. Given a candidate set G(*s*), (10) partially orders this set by considering the Boolean Complexity of the meanings of its elements; this ordering, in turn, predicts relative processing costs when the hearer selects one or other element from G(*s*). However, (10) says nothing about how the cost of processing an element in G(*s*) would relate to the cost of processing a form-meaning pair outside of this set. Note also that the measure is context-invariant and that it does not reference the computational history of the elements of G(*s*). All that matters is what the different meanings in G(*S*) are.

**Figure 1:**
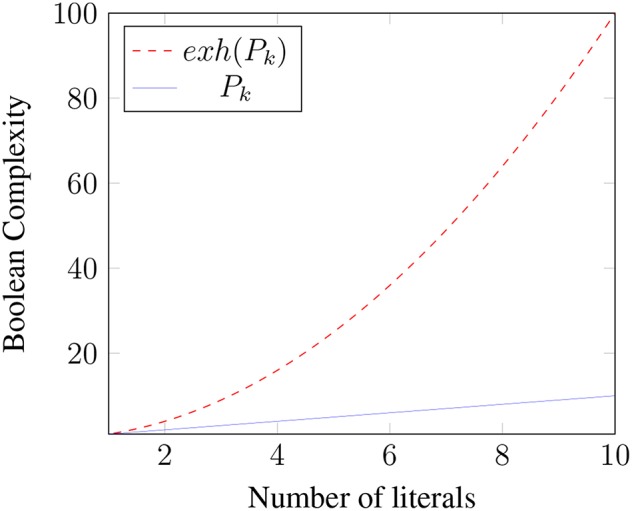
Growth of Boolean complexity.

The relative complexity of an exhaustified binary disjunction extends to Boolean *k*-space for any *k*. To simplify our discussion of the general case, first note that in the binary case [[*exh*(*p* ∨ *q, ALT*(*p* ∨ *q*))]] = (*p* ∨ *q*) ∧ ¬(*p* ∧ *q*) ⇔ (*p* ∧ ¬*q*) ∨ (¬*p* ∧ *q*) = [[*exh*(*p, C*) ∨ *exh*(*q, C*)]], where *C* = {*p, q*}. More generally, where *P*_*k*_ is a *k*-ary disjunction *p*_1_ ∨ *p*_2_ ∨ … ∨ *p*_*k*_ and *C* = {*p*_1_, …, *p*_*k*_}, it is easily shown that [[*exh*(*P*_*k*_, *ALT*(*P*_*k*_))]] = [[*exh*(*p*_1_, *C*) ∨ …*exh*(*p*_*k*_, *C*)]] (i.e., “only *p*_1_” or “only *p*_2_” or … “only *p*_*k*_”)^11^. This meaning can be expressed as the disjunction of *k* propositions, each of which is a conjunction of *k* literals in which one literal is positive and the rest are negative: (*p*_1_ ∧ ¬*p*_2_ ∧ …∧¬*p*_*k*_)∨(¬*p*_1_∧*p*_2_∧¬*p*_3_∧…¬*p*_*k*_)∨…(¬*p*_1_∧…∧¬*p*_*k*−1_∧*p*_*k*_). Thus, exhaustification of *P*_*k*_ not only strengthens the meaning of *P*_*k*_, but it also creates a more *complex* meaning by converting a proposition with complexity *k* to one with complexity *k*^2^. Figure 1 illustrates how the Boolean Complexities of *P*_*k*_ and *exh*(*P*_*k*_) grow with *k*.

The Boolean Complexity perspective might thus provide a motivation for having *exh* in the first place. For note that *exh* allows speakers and hearers to convey relatively complex meanings by uttering relatively simple formulae. For example, *exh* allows speakers and hearers to use, say, a disjunction of 10 literals (hence complexity 10) to convey a message with 10 times that complexity: *B*([[*exh*(*P*_10_)]]) = 100. Of course, the application of *exh* also increases syntactic complexity (if we identify *STR* with *exh*), and the code for *exh* needs to be stored and executed. All of this will induce some cost. The tradeoff is presumably such that it is nevertheless an improvement on having to actually utter the more complex formula that would be required without *exh*.

Even if *exh* may have been “designed” in part to produce higher-complexity meanings from simpler ones, it does not always do so. For example, recall that under certain conditions *exh* can produce a *conjunctive* strengthening of a disjunctive sentence. Recall also that in such cases there appears to be no corresponding cost associated with *exh*. The Boolean Complexity analysis provides at least a partial answer to this: since conjunction and disjunction have the same Boolean Complexity, there is no expected cost under (10) when *exh* turns a disjunctive basic meaning into a conjunctive strengthened meaning^12^.

Significant challenges remain. First, (10) does not speak to why conjunctive inferences should be *less costly* than their literal counterparts. Chemla and Bott (2014) found that—unlike scalar implicatures like “some but not all”—free-choice inferences are faster than their literal counterparts. They also found that—again unlike scalar implicatures like ‘some but not all”—the rate at which free-choice inferences are selected does not drop under time constraints. As they put it (Chemla and Bott, 2014, p.392): “*not* deriving a free choice inference is a costly phenomenon.” Furthermore, not only are conjunctive inferences less costly than their literal competitors, there appears to be a substantial *preference* to select the conjunctive reading when it is available (e.g., Chemla, 2009b; Bowler, 2014; Chemla and Bott, 2014; Meyer, 2015; Singh et al., 2016b; Bar-Lev and Fox, 2017; Tieu et al., 2017). In fact, even in concept learning, it is an old observation that conjunctive concepts are easier to acquire than disjunctive concepts. Thus, in both concept learning and in exhaustification, the order of difficulty appears to be the same:

(11) Cognitive difficulty of connectives: Conjunctions are easier than inclusive disjunctions which in turn are easier than exclusive disjunctions.

Boolean Complexity tells us why exclusive disjunctions are harder than inclusive disjunctions, but it does not tell us why inclusive disjunctions are harder than conjunctions. We will address this challenge in section 3. Before we do that, note that (10) is limited to propositional sentences. We need a general metric that could apply to quantified sentences as well. This would allow us to replace “Boolean Complexity” with a more general notion of “semantic complexity”. We discuss this in the next section.

### 2.2. Semantic Automata

Consider sentences *QAB*, where *Q* is a quantifier, *A* its restrictor, and *B* its scope. Well-known constraints on natural language quantifier denotations allow us to view quantifiers as machines that determine acceptance/rejection based on two inputs only: those *A* that are *B* and those *A* that are not *B* (van Benthem, 1986). Call the first kind of input “1” and the latter “0.” Given this perspective, quantifiers can be viewed as computational devices that accept certain strings over the alphabet {0, 1}. Call the set of strings accepted by the machine corresponding to quantifier *Q* the *language* accepted by *Q*, L(*Q*).

In the cases of interest to us, such as *some* and *all*, the quantifiers correspond to the simplest kinds of computing devices, namely finite-state-machines^13^. For example, a quantifier like *some* will accept any string as long there is at least one 1 in it (i.e., as long as there's at least one *A* that's a *B*). Here is a diagram of a machine that does this:

(12) Automaton accepting *some*: 
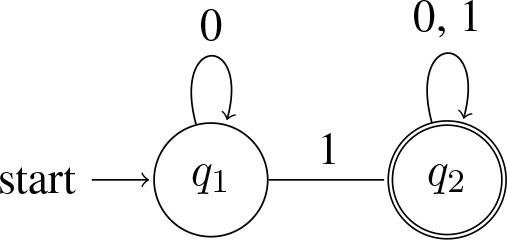

In words, the machine starts in the start state *q*_1_, and it processes the string one symbol at a time in left-to-right order. The arrows determine what the machine does upon processing a symbol. If it sees a 0 in state *q*_1_, it remains in *q*_1_ and moves on to the next symbol. If it sees a 1 in state *q*_1_, it moves to state *q*_2_ and moves on to the next symbol. Once in *q*_2_, it remains there—neither a 0 nor a 1 can get it out of *q*_2_. When all symbols in the string have been processed, the machine accepts the string if it is in an ‘accept' state when the string ends; otherwise, it rejects the string^14^. In our diagram, *q*_2_ is the ‘accept' state, marked by double-circles.

Inspection of the machine in (12) at once reveals that it accepts strings like 1, 01, 000010101, 111, and that it rejects strings like 0, 000, and 00000. More generally, the language accepted by ∃ is L(∃) = {*w* : *w contains at least one* 1}.

A quantifier like *all*, on the other hand, will reject a string as soon as it processes a single 0 (a single *A* that is not a *B*). That is, it accepts strings that contain only 1s: L(∀) = {*w* : *w* = 1^*n*^ for *n* > 0}^15^. Here is a machine that accepts L(∀) (note that in this machine, *q*_1_ is both the start state and accept state):

(13) Automaton accepting *all*: 
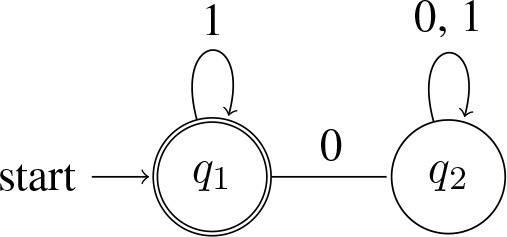

Given this formal apparatus, we can associate a quantifier *Q*'s semantic complexity with the size of the smallest machine that accepts L(*Q*):

(14) Quantifier complexity:
  - a. The semantic complexity of a quantifier *Q* is the *minimum* size finite-state-machine that accepts L(*Q*).
  - b. The *size* of a machine is the number of states in the machine.

The machines in (12) and (13) are equally complex: they each have two states, and no smaller machines can be constructed that accept their respective languages. Note also that this definition of complexity is independent of the details of the syntactic expressions used to convey these meanings.

Now, recall that among the elements that L(∃) accepts are strings like 11, 111, 1111, etc. These of course are the strings accepted by L(∀). The semantic notion of entailment is realized here as a subset relation over bit strings: L(∀) ⊆ L(∃). Application of *exh* breaks the entailment: *exh*(∃) = ∃^+^ = ∃∧ ¬∀, and L(∃^+^) = {*w*:*w* contains at least one 0 and at least one 1}. Here is a machine that accepts this language:

(15) Automaton accepting *some but not all*: 
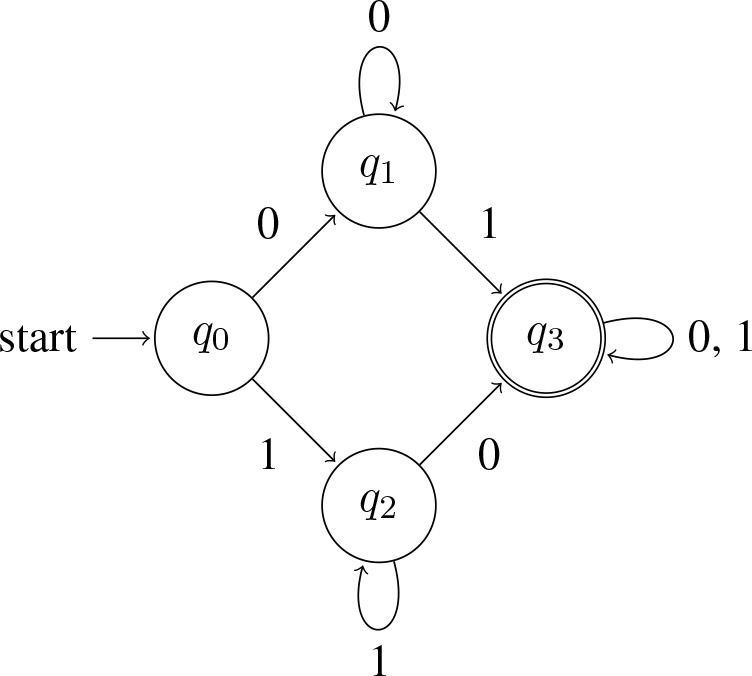

This machine is more complex than the ones in (12) and (13) (four states vs. two). Intuitively speaking, the additional complexity arises because determining membership in L(∃^+^) is a more demanding task. At any given point, a machine has to be ready to answer ‘yes' or ‘no.' Its memory is finite, but it does not know how long the input string is. Thus, the machine needs strategies for keeping track of relevant information without having to store the entire history of the string. The machine corresponding to ∃ in (12) needs to keep track of whether it has seen a 1 yet (if so, accept; otherwise, reject). The machine corresponding to ∀ in (13) needs to keep track of whether it has seen a 0 yet (if so, reject; otherwise, accept). The machine corresponding to ∃^+^ in (15) needs to keep track of *both* of these pieces of information: it needs to keep track of whether it has seen a 1 yet and it needs to keep track of whether it has seen a 0 yet. The machine accepts the string only if the answer to both questions is “yes,” but there are different paths to this state: one begins by having seen a 0 first, in which case the machine's strategy is to wait for a 1 and answer “yes” if and only if it encounters one, and the other begins by having seen a 1 first, in which case the machine's strategy is to wait for a 0 and answer “yes” if and only if it encounters one.

There is prior evidence that a quantifier's complexity has detectable psychological correlates. For example, recent evidence from implicit learning tasks suggests that concepts whose membership is determined by ∀ are preferred to those whose membership is determined by ∃^+^ (Buccola et al., 2018). Like the relative ease of learning conjunctive concepts over exclusive disjunction concepts, considerations of semantic complexity would appear to provide a natural account for this finding^16^. From a different direction, Szymanik and Thorne (2017) present evidence that the frequency of a quantifier's occurrence is to some extent predictable from its semantic complexity.

It is plausible, then, to think that quantifier complexity might also be a relevant factor in parsing costs. In particular, it might provide a rationale for why application of *exh* to ∃ tends to be costly: the meaning ∃^+^ is inherently more complex than ∃ and is thus cognitively more demanding. Like with Boolean Complexity, the parser pays a penalty for choosing a complex meaning even though a simpler one was available.

(16) Quantifier complexity and processing costs during disambiguation: Let *S*_*Q*_ be a sentence containing quantifier *Q*, and suppose that the grammar G assigns *k* analyses to *S*_*Q*_: G(*S*_*Q*_) = {λ_1_, …, λ_*k*_}, where each λ_*i*_ is a form-meaning pair < *f*_*i*_, *m*_*i*_ >. Let *Q*(*m*) be the Quantifier Complexity of meaning *m*. Then the *cost of selecting* λ_*i*_ ∈ G(*S*), *C*(λ_*i*_), is proportional to the Quantifier Complexity of meaning *m*_*i*_: *C*(λ_*i*_) ∝ *Q*(*m*_*i*_).

Given this definition, we will now simply use the term “semantic complexity” to refer to whichever of (16) or (10) applies, letting context choose.

Like with (10), the statement in (16) explains only some of the relevant facts. For example, consider numerals. A sentence like *Sandy ate two apples* on its basic meaning conveys that Sandy ate at least two apples. Its strengthened meaning is that Sandy ate exactly two apples. The strengthened meaning is not only stronger, but also more complex^17^:

(17) Machine accepting *at least 2* 
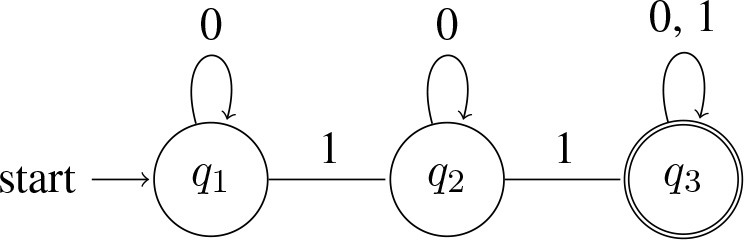

(18) Machine accepting *exactly 2* 
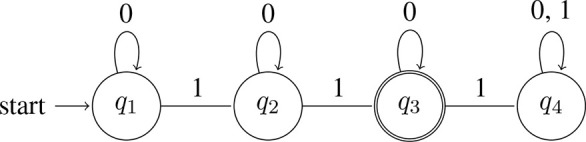

Despite this additional complexity, as we discussed earlier (section 1.3), the strengthened meanings of numerals are nevertheless easy to process and are often preferred to their unstrengthened counterparts. Furthermore, in concept learning the propositional results appear to carry over to their quantificational analogs. Specifically, it appears that when the data are consistent with ∀ and with ∃, learners tend to conclude that the underlying rule is universal rather than existential (Buccola et al., 2018). Clearly, semantic complexity cannot explain this^18^.

In propositional and quantificational sentences, then, semantic complexity appears to provide at best a partial account of the relevant facts. In particular, it appears to explain cases where a disjunctive operator like ∨ or ∃ is strengthened by negating conjunctive alternatives like ∧ or ∀, respectively. In such cases the result is a more complex meaning. In these cases, there is no CUPID: when the competence system applies *exh*, it creates a more complex syntactic object with a more complex meaning, and this complexity is realized in performance with a cost. It would make sense for there to be pressures to avoid this additional complexity if possible, and for there to be costs for selecting the more complex form-meaning pair against simpler alternatives. As noted, this pressure appears to be present in concept learning exercises as well: it is easier for participants to acquire a ∀/∧ -concept than a ∃^+^/∨ ^+^ concept.

However, semantic complexity does not speak to why ∀/∧ -concepts are easier to learn/process than their ∃/∨ variants. And semantic complexity does not explain CUPID: free-choice inferences and exactly-readings of numerals are less costly than their basic meaning counterparts even though they are not semantically simpler than them. Clearly, it can't be that semantically *stronger* meanings are less costly than their weaker basic meaning counterparts, given that ∃^+^/∨ ^+^ are stronger than ∃/∨ but are nevertheless harder to learn/process. The CUPID problem is still with us.

## 3. Questions, Answers, Contexts, and Processing Costs

The above complexity measures provide an *a priori*, context-invariant ordering of meanings that the agent may apply before they have learned anything. As the agent accumulates information, and as the common grounds of their conversations become richer, these language-external domains will begin to exert a greater influence on parsing and interpretation strategies, and may in some cases counter the *a priori* orderings the organism starts with. I will argue that the solution to CUPID involves considerations of how candidate meanings interact with the context of use. On the classic Stalnakerian picture, sentences are uttered and understood in context, and sentences update the input context in rule-governed ways to create a new output context relative to which the next utterance will be interpreted. Thus, contexts and sentences have a dynamic interplay that we will momentarily exploit to help us overcome the limitations of semantic complexity alone.

Specifically, I will argue (building on Singh et al., 2016b) that the extent to which a given meaning resolves the question under discussion (QUD) is a predictor of the costs of accepting it into the common ground. The better the answer, the lower the cost. Here, “goodness” is a function of how close to a complete answer the meaning provides, i.e., how close it comes to locating the one true cell in the partition induced by the QUD. I motivate this idea briefly in section 3.1, and show in section 3.2 that the parsing mechanism proposed in Singh et al. (2016b) provides the pieces needed to overcome the problem posed by numerals and free-choice inferences. That system included two interacting constraints that were evaluated by an Optimality-Theoretic system: (i) a constraint that penalizes incomplete answers (considered as semantic objects), (ii) a constraint that penalizes syntactic complexity (occurrences of *exh*). In section 2, I proposed a way to replace (ii) with a measure of semantic complexity, and in section 3.2, I show how to incorporate this amendment into the system in Singh et al. (2016b).

In sections 3.3 and 3.4, I further modify Singh et al.'s (2016b) proposal by changing the way processing costs relate to answers. Specifically, Singh et al. (2016b) suggested that complete answers have no cost but partial answers do, and that partial answers are equally costly. In section 3.3, I will motivate the idea that partial answers can be ordered for quality by how far they are from complete. I also review and reject some simple options for formalizing this distance, and in section 3.4 I provide a domain-general way to measure distance using the information-theoretic concept of entropy (Shannon, 1948). Entropy has a well-known compression interpretation (number of bits needed to eliminate the uncertainty), thus making it plausible that both semantic complexity and entropy have a compression-related cost. I will suggest that this lends flexibility in formulating functions that combine these costs. For example, it allows us to abandon the OT evaluation system and instead use simple arithmetic. Here is my proposal:

(19) Processing costs during disambiguation: Let *S* be a sentence uttered in context *c*. Suppose that grammar G assigns *k* analyses to *S*: G(*S*) = {λ_1_, …, λ_*k*_}, where each λ_*i*_ is a form-meaning pair < *f*_*i*_, *m*_*i*_ >. Let S(*m*_*i*_) be the semantic complexity of *m*_*i*_, let *c*_*i*_ be the result of updating context *c* with *m*_*i*_, *c* + *m*_*i*_, and let H(*c*_*i*_) be the entropy in context *c*_*i*_. Then the *cost of selecting* λ_*i*_ ∈ G(*S*) in context *c*, *C*(λ_*i*_, *c*), is: *C*(λ_*i*_, *c*) = S(*m*_*i*_) + H(*c*_*i*_).

We will now build our way to the cost function in (19), highlighting various choice points as we go. We begin with the importance of questions and answers and more generally with the way normative demands on speech might play a role in processing costs.

### 3.1. Norms of Good Conversational Behavior and Processing Costs

It is commonly assumed that there are *normative* demands on a speaker, such as the demand that they be truthful, informative, relevant, assert things they have evidence to support, use sentences whose presuppositions are satisfied (or easily accommodated), among other constraints on their behavior (e.g., Grice, 1967; Stalnaker, 1978; Williamson, 1996, and much other work). Listeners pay attention to whether these demands are satisfied. There are consequences when it is detected that a speaker misbehaved according to these norms. There is surprise, embarrassment, hostility, and trust and credibility are broken. These considerations suggest that the maxims should be viewed as rules of decent cooperative behavior, which in particular apply even when it is in the speaker's interest to violate them. A speaker may decide, for instance, to speak a falsehood or omit relevant damning information, but even if this maximizes their utility in some sense this would not justify their action. They are held to the maxims independent of the utility of their doing so. All else being equal, then, we assume that a speaker is more likely to be obeying the norms than violating them.

(20) Assumption about language use: Unless we have reason to think otherwise, assume that a speaker is obeying conversational maxims.

If (20) is a true assumption about conversation, we would expect it to be relevant to disambiguation. In particular, suppose that λ_1_ and λ_2_ are competing form-meaning pairs, and that λ_1_ violates a norm of language use and λ_2_ does not. We would expect (20) to generate a pressure in favor of λ_2_. It is of course hard to tell whether someone is speaking truthfully, or has evidence to support what they assert. But it is easy to tell whether a speaker is being *relevant*^19^. Specifically, suppose that the ideal speaker is assumed to be optimally relevant, by which we mean that they immediately (when it's their turn to speak) settle the Question Under Discussion (QUD). Assume further that QUDs can be modeled as partitions of the common ground (e.g., Groenendijk and Stokhof, 1984; Lewis, 1988, among others). For example, *PART*(*c*) = {*pq, pq*′, *p*′*q, p*′*q*′} is a partition that divides *c* into four sets of worlds (cells of the partition): those where *p* and *q* are both true (*pq*), those where *p* is true and *q* is false (*pq*′), those where *p* is false and *q* is true (*p*′*q*), and those where both *p* and *q* are false (*p*′*q*′). An *answer* is a union of cells, and a *complete answer* is a particular cell.

What we want in a context is a complete answer. If I ask you who was at the scene of the crime, and you know the answer (‘the whole truth'), you are required to tell me. Given any proposition *r* asserted by the speaker, we can readily examine whether *r*—together with the information in the common ground—identifies a cell. That is, we can readily answer the question: ∃*u* ∈ *PART*(*c*) : *u* = *r* ∩ *c*? If the answer is positive, the listener will be satisfied that the question has been resolved. Otherwise, the speech act will have left undesired relevant uncertainty. This goes against our expectation that the speaker would fulfill their obligations, at least if they don't flag that they are unable to do so.

Thus, consider the following principle proposed in Singh et al. (2016b)^20^.

(21) Complete Answer Preference: If there is an analysis λ_*i*_ = < *f*_*i*_, *m*_*i*_ > of sentence *S* such that *m*_*i*_ completely answers the QUD in *c*, then—all else being equal and assuming no other candidate completely answers the QUD—λ_*i*_ will be preferred.

Suppose, then, that the parsing mechanism encodes an expectation that the speaker is obeying all relevant maxims. The parser will therefore expect to find among the form-meaning pairs provided by the grammar one that will completely answer the QUD (among other demands on good conversational behavior). If it finds one, then it will select it and no cost is induced. They have simply applied their grammatical principles to analyze the sentence and their normative expectations have been satisfied. However, something goes wrong if the QUD is not completely answered. The listener will be surprised, and other considerations might enter into disambiguation decisions and therefore also into the consequences of these decisions.

### 3.2. The Parsing Proposal in Singh et al. (2016b) With Semantic Complexity in Place of Syntactic Complexity

Singh et al. (2016b) suggested an Optimality-Theoretic processing mechanism that incorporated a preference for a complete answer and a pressure against syntactic complexity. Specifically, the system posited (i) a high-ranked constraint ^*^INC that penalizes form-meaning pairs that fail to provide a complete answer to the QUD, and (ii) a low-ranked constraint ^*^exh that penalizes a form-meaning pair for each occurrence of *exh* in the parse. In that system, when no form-meaning pair provides a complete answer to the QUD, considerations of syntactic complexity (approximated by number of occurrences of *exh*) adjudicate between the remaining candidates. By ranking ^*^INC above ^*^exh, the system assumes that a sentence's ability to resolve relevant contextual uncertainty is worth any syntactic cost that might be incurred by adding *exh*. Furthermore, by positing ^*^exh, the system identified the number of occurrences of *exh* as a proxy for the sentence's complexity, and hence used the *form* of the sentence as its complexity measure.

In this paper I am pursuing the idea that the parser is only sensitive to the *meanings* of candidates. Thus, when no form-meaning pair provides a complete answer, the amendment needed in Singh et al. (2016b) would be to posit that *semantic* complexity determines the parser's choice. This could be implemented by replacing ^*^exh with ^*^SC (for “semantic complexity”), and by assigning a candidate form-meaning pair a number of violations equal to its semantic complexity. Here we show that this amendment captures all the facts that Singh et al.'s (2016b) proposal was designed to account for, and that the constraint ^*^INC accounts for CUPID under the assumption that it is higher-ranked than ^*^SC.

Consider again the question faced by a listener about whether or not to exhaustify the input sentence. Suppose that a disjunctive sentence like *p* ∨ *q* is uttered in response to a (possibly implicit) QUD like *which of p and q is true*? That is, suppose it is uttered in a context in which the partition is *PART*(*c*) = {*pq, pq*′, *p*′*q, p*′*q*′}^21^. Of course, a disjunctive answer *p* ∨ *q* only gives a partial answer, eliminating just the cell *p*′*q*′. A better answer is made available by *exh*: in the adult state *exh*(*p* ∨ *q*) would also eliminate the cell *pq*. This is better—it generates fewer ignorance inferences than the parse without *exh* (Fox, 2007)^22^. However, it is still an undesirable and unexpected state of affairs because it continues to leave us with relevant uncertainties. In fact, as noted in Singh et al. (2016b), we appear to have prosodic contrasts between complete and partial answers, but not between better and worse partial answers. This observation indicates that what matters for answerhood—at least so far as prosody is telling—is whether the sentence provides a path to a complete answer. In the adult state with plain disjunctive sentences, the parser has no analysis available to it that provides it with a complete answer. In such a case, ^*^SC will get a chance to decide the optimal analysis. Here, the *a priori* ordering between the simpler inclusive disjunction and the more complex exclusive disjunction (cf. section 2.1) would pressure against the exclusive disjunction. Assuming that less optimal candidates are costlier than optimal candidates, we predict the observed cost for the exclusive disjunction reading of *A or B*.

(22) Strengthening inclusive disjunction to exclusive disjunction:

**Table:**
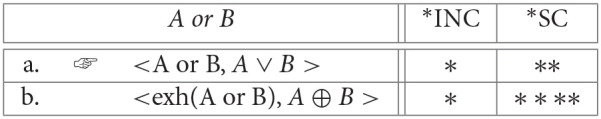


Things are different when disjunctive sentences have alternatives that are not closed under conjunction. In such cases, *exh* can turn the disjunction into a conjunction (Fox, 2007; Singh et al., 2016b; see also Chemla, 2009a; Franke, 2011; Bar-Lev and Fox, 2017 and note 4). Assume the treatment in Fox (2007) and Singh et al. (2016b) under which recursive application of *exh* turns *p* ∨ *q* into *p* ∧ *q*: [[*exh*^2^(*p* ∨ *q*)]] = *p* ∧ *q*. On the face of it one might have expected this computation to be hard, since there are multiple applications of *exh* and multiple sets of alternatives that get generated. However, recall that we are assuming that these computations do not contribute to costs. Instead, it is the *output* of these computations (^*^SC), and its affect on the context (^*^INC), that are relevant to processing costs. In this case, the parser finds the conjunctive meaning and considers it desirable because it provides a complete answer and no cost is therefore expected.

(23) Strengthening inclusive disjunction to conjunction:

**Table:**
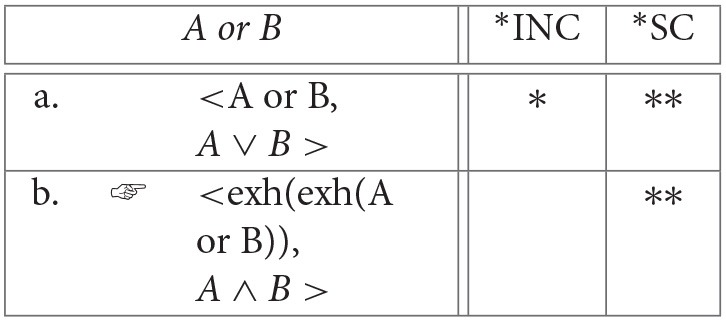


More generally, if it is reasonable to assume that a disjunction *P*_*k*_ = *p*_1_∨*p*_2_∨…*p*_*k*_ will typically be used in a context in which the participants are interested in knowing, for each of the disjuncts *p*_*i*_, whether *p*_*i*_ is true, then we have an explanation for the contrast between conjunctive strengthenings and exclusive strengthenings and their relative ordering with inclusive disjunction [cf. the generalization in (11) in section 2.1]: conjunctive readings satisfy the high-ranked ^*^INC whereas neither inclusive nor exclusive disjunctions do, and inclusive disjunctions have fewer ^*^SC violations than exclusive disjunctions.

The result extends to quantificational sentences *DAB*, where *D* is a quantificational determiner, *A* its restrictor, and *B* its nuclear scope. Suppose such sentences are typically used in answers to the question *How many A B?* If there are *k* individuals in the domain, then this induces a partition with *k* + 1 cells (“none,” “exactly 1,” “exactly 2,” …, “exactly k”). When *D* is a logical existential quantifier as in *some A B*, the basic meaning ∃ only eliminates the “none” cell. This partiality is expected, given that existential quantifiers are essentially disjunctive: “exactly 1 or exactly 2 or … or exactly *k*.” Exhaustification can produce a slightly better answer by eliminating the “exactly k” cell, but it still typically leaves you without the expected and desired complete answer because you are still left wondering which of exactly 1 or exactly 2 or … or exactly *k* − 1 is true. Thus, both ∃ and ∃^+^ violate ^*^INC. However, because ∃ is semantic simpler than ∃^+^ (2 vs. 4; cf. section 2.2), ^*^SC decides in favor of ∃ and ∃^+^ is therefore predicted to be costly.

If the question were one that induced a different partition, say {∃∧¬∀, ∀, ¬∃}, then the costs for *exh*(∃) could disappear because it would now satisfy the high-ranked ^*^INC and ∃ still would not (see Breheny et al., 2013 for evidence in this direction). This is a general feature of the proposal: the costs for processing any sentence *S* will depend on what the QUD is. Sometimes *exh* can help you turn *S* into a complete answer, in which case no cost is expected, but other times *exh* will only create more complex meanings without also creating a complete answer, in which case costs are expected^23^.

When *D* is a numeral, *exh* will typically produce a complete answer to a *how-many* question. Suppose that there are *k* individuals in the domain, and that the speaker produces *nAB* where *n* < *k*. On its basic meaning, *nAB* is again only a partial answer, eliminating all cells “exactly r” where *r* < *n*. Again, this is expected given that the basic meaning is essentially disjunctive: “either exactly *n* or exactly *s*(*n*) or … or exactly *s*^*j*^(*n*)” (where *s*^*j*^(*n*) = *k* and is the result of *j* = *k* − *n* applications of the successor function to *n*). But with numerals, unlike with logical *some*, *exh* can produce a complete answer by also eliminating cells “exactly r” where *n* < *r* ≤ *k* (because, following Horn, 1972, the alternatives for *n A B* include not just *k A B*, but also *r A B* for *n* < *r* ≤ *k*)^24^. For example, consider the case where *n* = 2. Refer to the basic “at-least” reading with [≥ 2], and to the strengthened “exactly” reading with [= 2]. Then the OT constraint evaluation system selects [= 2] as optimal because it satisfies ^*^INC, even though the “exactly” reading incurs more violations of the lower-ranked ^*^SC (cf. Note 17 in section 2.2):

(24) Strengthening numerals from an “at least” to an “exactly” reading:

**Table:**
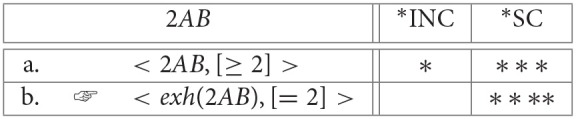


The system in Singh et al. (2016b) thus accounts for CUPID by appealing to the importance of complete answers in an overall theory of processing costs. The complete answer perspective may also speak to some of the questions that remain unanswered in concept learning. Recall that conjunctive concepts are easier to learn than inclusive disjunction concepts, and that universal quantification is easier to learn than existential quantification. We now have a rationale for this: if you learn that some element satisfies a conjunctive concept (say *red and triangle*), you learn right away that it is red and that it is a triangle. Disjunctive concepts—whether inclusive or exclusive—leave this question open. Similarly, learning that *All wugs are red* tells you that as soon as you encounter a wug, you can infer something about its color. Learning only that some wugs are red, or that only some wugs are red, does not confer you with this inferential ability. Presumably, as with conversation, it is better to have relevant uncertainties resolved than to leave them unresolved. Recall that these considerations cannot be reduced to considerations of semantic strength: for example, conjunction and exclusive disjunction are both stronger than inclusive disjunction, but only conjunction is easier to process.

### 3.3. Complete vs. Partial Answers

We have been assuming with Singh et al. (2016b) that the parser cares only about whether a given form-meaning pair provides a complete answer to the QUD. As we noted earlier, this assumption is motivated in part by the observation that our pronunciation patterns distinguish between complete and partial answers but not between different kinds of partial answer. An additional motivation comes from considerations of our obligations in general. If I ask my son to help me carry a stack of books from one room to the other, and the request is reasonable, I expect him to help me move all of them. I would be surprised and disappointed with anything less.

But what if he helped me move half of them and then went back to his video games? Is that not better than opting out entirely? The system in Singh et al. (2016b) treats all sub-optimal answers on a par. For example, in (22) both the inclusive disjunction and exclusive disjunction receive a single penalty for violating ^*^INC, even though the exclusive disjunction is a better answer (it rules out two cells instead of only one). Even if prosody is blind to this distinction, it is not obvious that the parsing mechanism should be. Some partial answers are closer to complete than others, and it is conceptually natural to think that the parser might care about how close different possibilities get to the end goal. To facilitate comparison with Singh et al.'s (2016b) binary choice (*complete or not?*), it would be useful to formulate a measure that allowed partial answers to be compared for how far they are from complete. Here we aim to find such a measure, and to examine its usefulness in accounting for the facts under discussion. Here I review some fairly simple measures, but I will reject them in favor of the information-theoretic entropy measure proposed in section 3.4. Readers may skip straight to the proposal there, but I provide details here because it might be instructive to see why arguably simpler proposals don't work.

One natural amendment of Singh et al. (2016b) that could accommodate the ordering assumption would be to count the number of remaining cells in the partition and to use that as the number of ^*^INC violations (1 being the minimum value associated with the complete answer). Call our new constraint ^*^INC-*G* (where *G* is for “graded”). Under this view, conjunctions would still be optimal when compared with inclusive disjunctions: they identify a unique cell, whereas disjunctions leave three cells to choose from.

(25) Strengthening inclusive disjunction to conjunction:

**Table:**
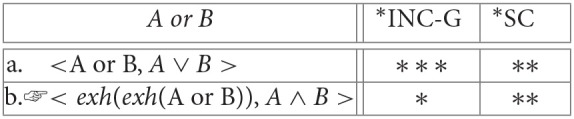


Unfortunately, the move from ^*^INC to ^*^INC-*G* quickly runs into trouble. For example, exclusive disjunctions come out as optimal in competition with inclusive disjunctions because they only leave behind two cells:

(26) Strengthening inclusive disjunction to exclusive disjunction:

**Table:**
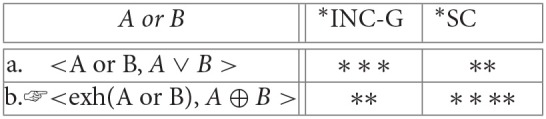


This is the wrong result. We could correct for this by actually reordering the constraints such that ^*^SC outranks ^*^INC. This would work for (26) and for (25), but it would not work for numerals. For example, if the sentence *2* *AB* is offered in response to the question *how many (of these 4) As are B?*, the evaluation component would select the basic “at-least” reading as optimal:

(27) Strengthening numerals from an “at least” to an “exactly” reading:

**Table:**
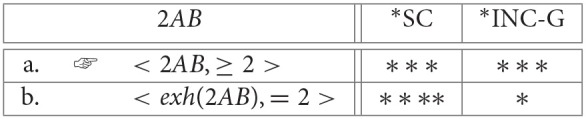


These considerations could of course be taken as an argument that the parser does not after all distinguish between different kinds of partial answer, and thus that the parsing mechanism incorporates ^*^INC instead of ^*^INC-*G* and orders ^*^INC over ^*^SC. The challenge for this view would be to provide a rationale for why the constraints should be ordered in this way.

In the rest of this paper I will continue to take a different path so that we have a concrete viable alternative that allows room for orderings of partial answers. As a starting point, suppose that the problem is not with ^*^INC-*G* but with the OT evaluation system. Specifically, assume that costs are equated with the total number of constraint violations. Different cost functions are imaginable, but let us take summation as a simple starting point. Under this view, it turns out the above facts can all be captured. For example, in the case of binary connectives, conjunctions are less costly than inclusive disjunctions (three vs. five) which in turn are less costly than exclusive disjunctions (six). Similar results hold for quantified sentences. Suppose that there are *k* individuals in the domain. Then ∃ costs *k*+2 and ∃^+^ costs *k*+3: ∃ incurs two violations of ^*^SC and *k* violations of ^*^INC-*G* (it only eliminates the cell in which no individuals that satisfy the restrictor satisfy the scope, leaving behind *k* cells), and ∃^+^ incurs four violations of ^*^SC and *k*−1 violations of ^*^INC-*G* (it also eliminates the cell in which all individuals that satisfy the restrictor satisfy the scope). Finally, numerals *nAB* (where *n* < *k*) are also accounted for: the “at-least” reading has *n*+1 violations of ^*^SC and (*k* − *n*) + 1 violations of ^*^INC-*G*, and hence *k*+2 violations in total, whereas the “exactly” reading has *n*+2 violations of ^*^SC and one of ^*^INC-*G*, for *n* + 3 violations in total. For all values of *n* and *k* such that *n* < *k*, the “exactly” reading is no more costly than the “at least” reading, and for all but the case *k* = *n* + 1 the “exactly” reading is less costly.

Unfortunately, this perspective leads to some counter-intuitive predictions. Consider the case of a general *k*-ary disjunction *P*_*k*_, and consider the costs associated with the basic meaning of the sentence, as well as with *exh*(*P*_*k*_) (leading to the “only one” reading) and with $exh^{2}\left(P_{k}\right)$ (leading to the conjunctive reading when the alternatives are not closed under conjunction; see note 4). Recall from section 2.1 that the semantic complexity of *P*_*k*_ is *k* (the smallest formula representing this meaning is *p*_1_ ∨ *p*_2_ ∨ … ∨ *p*_*k*_), which is also the semantic complexity of $exh^{2}\left(P_{k}\right)$ because this gives the incompressible *p*_1_ ∧ *p*_2_ ∧ …*p*_*k*_. Recall also that *exh*(*P*_*k*_) is more complex: its meaning is given by *k* disjuncts each of which contains *k* conjuncts that assert that one of the *p*_*i*_ is true and all other *k* − 1 *p*_*j*_ are false. Thus, *exh*(*P*_*k*_) has semantic complexity *k*^2^. We also need to say something general about how these meanings affect the QUD. With *k* literals, there are 2^*k*^ cells of the partition. Conjunctions completely answer the QUD, and hence leave behind a single cell in which each literal *p*_*i*_ in *P*_*k*_ is true. Inclusive disjunctions *P*_*k*_ eliminate only the cell in which all literals *p*_*i*_ in *P*_*k*_ are false, and hence they leave behind 2^*k*^ − 1 cells. Finally, *exh*(*P*_*k*_) leaves behind *k* cells in each of which only one of the literals *p*_*i*_ in *P*_*k*_ is true. We summarize these costs in (28):

(28) Costs of update (to be revised):

**Table:** 

Formula	^*^SC	^*^ING-*G*
*P*_*k*_	*k*	2^*k*^ − 1
*exh*(*P*_*k*_)	*k*^2^	*k*
*exh*^2^(*P*_*k*_)	*k*	1

**Figure 2:**
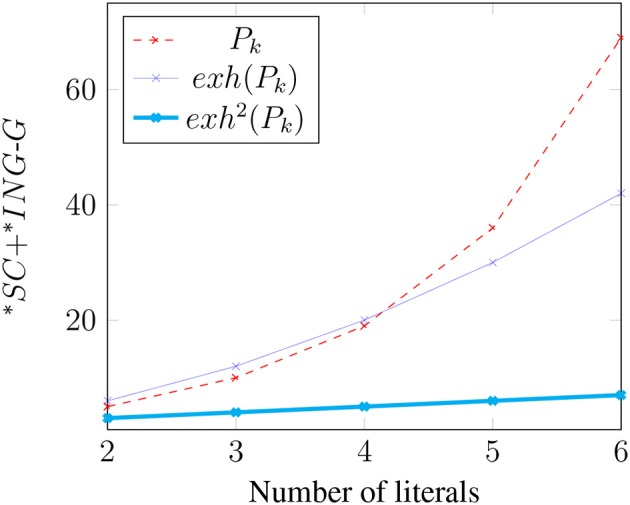
Cost of update (to be revised).

Continue to assume that costs are simply added together. The cost of the conjunctive reading grows linearly with *k* (it is the sum *k* + 1), and thus still comes out less costly than the disjunctions because their costs grow more rapidly: *exh*(*P*_*k*_) grows as a polynomial *k*^2^ + *k*, and *P*_*k*_ grows exponentially *k* + 2^*k*^ − 1. The competition between the two disjunctions thus boils down to how quickly *k*^2^ grows vs. 2^*k*^ − 1. It turns out that exclusive disjunctions are predicted to be slightly more costly than inclusive disjunctions for 2 ≤ *k* ≤ 4 (in this range 2^*k*^ − 1 < *k*^2^), after which point the costs of inclusive disjunctions start to increasingly dwarf the costs of exclusive disjunctions (here 2^*k*^ − 1 ≫ *k*^2^). See Figure 2 for an illustration.

It would be surprising, hence interesting, if this prediction were true. But it seems rather unlikely. A more natural result would be one under which inclusive disjunctions are truly sandwiched between exclusive disjunctions and conjunctions for all values of *k*. Certainly, this is what all the evidence would suggest (Feldman, 2000). The problem, clearly, is the exponential cost associated with inclusive disjunctions because of the poor job they do at answering questions. They eliminate only one among an exponential space of cells, and they therefore leave behind an exponentially large amount of relevant uncertainty.

### 3.4. Entropy, Questions, and Answers

There is a natural perspective that tames the costs associated with exponential relevant uncertainty (van Rooij, 2004, building on Bar-Hillel and Carnap, 1952 among other work). Suppose that we identify relevant uncertainty with the *entropy* of a partition, which measures the amount of information a receiver would expect to receive from observing an outcome of this partition. Your relevant uncertainty is eliminated when you observe a given outcome, and the entropy of the partition therefore provides a natural measure of the amount of relevant uncertainty you started with. Clearly, the greater the number of alternatives we are considering, the more relevant uncertainty there is and hence the more informative any particular outcome would be. We thus want a measure of relevant uncertainty that is monotonically increasing in the number of cells. Simply counting the number of cells provides such a measure but as we saw it runs into trouble. Note also that the count measure makes no use of probabilities. For example, there is a sense in which a less likely cell is more informative than a more likely one. There is also a sense in which we are most uncertain if all cells are equally likely.

To account for these and other desiderata, Shannon (1948) argued that the information associated with any given cell *q*_*i*_ in partition Q should be identified with *log*(1/*P*(*q*_*i*_)), where *P*(*q*_*i*_) is the probability that *q*_*i*_ is the answer to the question (the message that we receive). From this, the relevant uncertainty of the partition is identified with its *entropy*, which in turn is just the expected information (the sum of the information provided by each cell weighted by its probability)^25^:

(29) Entropy and Information: Let *Part*(*c*) = Q = {*q*_1_, …, *q*_*k*_}. Let *P*(*q*_*j*_) be the probability of *q*_*j*_. Then:
  - a. Expected information: The entropy of Q, *H*(Q), is the expected information $H\left(Q\right)=\overset{k}{\underset{j=1}{\sum }}P\left(q_{j}\right)inf\left(q_{j}\right)$^26^.
  - b. Information: The information received from any particular cell *q*_*j*_ is *inf*(*q*_*j*_) = *log*_2_(1/*P*(*q*_*j*_)).

(30) Examples:
  - a. Let Q = {11, 10, 01, 00}, and suppose that the elements in Q have the same probability: ∀*q*_*j*_ ∈ Q:*P*(*q*_*j*_) = 1/4. Then for all *q*_*j*_ ∈ Q, *inf*(*q*_*j*_) = *log*_2_(4) = 2, and *H*(Q) = 2.
  - b. Let Q = {11, 10, 01, 00}, and suppose that the elements in Q have the following probabilities: *P*(11) = 1/8, *P*(10) = *P*(01) = 1/4, *P*(00) = 3/8. Then *inf*(11) = 0.53, *inf*(10) = *inf*(01) = 0.5, *inf*(00) = 0.375, and thus *H*(Q) = 1.9.
  - c. Let Q = {111, 110, 101, 100, 011, 010, 001, 000}, and suppose that the elements in Q have the same probability: ∀*q*_*j*_ ∈ Q:*P*(*q*_*j*_) = 1/8. Then for all *q*_*j*_ ∈ Q, *inf*(*q*_*j*_) = *log*_2_(8) = 3, and *H*(Q) = 3.

The examples in (30) indicate some general properties that motivate the entropic measure of relevant uncertainty. When the elements of a partition Q have the same probability (∀*q*_*i*_ ∈ Q:*P*(*q*_*i*_) = 1/|Q|), the entropy is the log of the size of the set: *H*(Q) = *log*_2_(|Q|). This makes sense: each cell *q*_*i*_ provides information *log*_2_(1/*P*(*q*_*i*_)) = *log*_2_(1/(1/|Q|)) = *log*_2_(|Q|), and since each cell is equally likely, *log*_2_(|Q|) is the amount of information we expect to receive. Note also that the partition induced by considering whether *k* literals are true has entropy *k* when all cells are equally probable. Thus, when there are more literals, and hence more cells in the partition, there is more uncertainty. Finally, note that the entropy is reduced when probabilities are not equal (you are most uncertain when you have no bias among alternatives).

Assume now that the cost associated with relevant uncertainty in a context is identified with the information-theoretic entropy of the QUD in that context. Assume also (to keep calculations simple) that the cells in the partition have equal probability^27^. The logarithmic growth of entropy means that the corresponding cost functions are now more contained.

(31) Costs of update (final version):

**Table:** 

Formula	Complexity	Entropy
*P*_*k*_	*k*	*log*_2_(2^*k*^ − 1)
*exh*(*P*_*k*_)	*k*^2^	*log*_2_*k*
*exh*^2^(*P*_*k*_)	*k*	0

**Figure 3:**
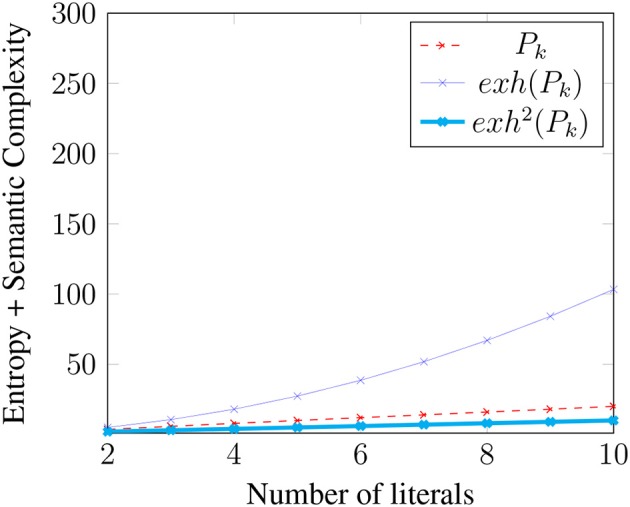
Cost of update (final version).

More to the point, we now predict the desired result that for all values of *k*, conjunctions are less costly than inclusive disjunctions which in turn are less costly than exclusive disjunctions (see Figure 3).

### 3.5. How Many Kinds of Cost?

With (31), we have completed our development of the cost function we stated in (19). We repeat the statement below in (32):

(32) Processing costs during disambiguation: Let *S* be a sentence uttered in context *c*. Suppose that grammar G assigns *k* analyses to *S*: G(*S*) = {λ_1_, …, λ_*k*_}, where each λ_*i*_ is a form-meaning pair < *f*_*i*_, *m*_*i*_ >. Let S(*m*_*i*_) be the semantic complexity of *m*_*i*_, let *c*_*i*_ be the result of updating context *c* with *m*_*i*_, *c*+*m*_*i*_, and let H(*c*_*i*_) be the entropy in context *c*_*i*_. Then the *cost of selecting* λ_*i*_ ∈ G(*S*) in context *c*, *C*(λ_*i*_, *c*), is: *C*(λ_*i*_, *c*) = S(*m*_*i*_)+H(*c*_*i*_).

At first blush, the two kinds of cost seem different. Semantic complexity is a measure of compressibility: what is the smallest representation that can produce the desired meaning? Entropy is a measure of relevant uncertainty: how much information is needed to resolve our uncertainty? As it happens, entropy has a coding interpretation. Shannon (1948) noted that the entropy tells us the length of the representation (in bits) that would be needed to communicate outcomes in Q^28^. Thus, both S and H give compression-based costs: semantic complexity tells us how much cost we have to pay for the current message, and entropy tells how much it would cost to get to a complete answer and hence how much cost we can expect to pay before our work is done^29^.

We may also want a more general variant of (32) that allows for other kinds of costs to be incorporated, and for different ways of combining them. For example, it is natural to consider the possibility that the information of a given answer might itself have a cost, or that entropy reduction (the difference in entropy between the input and output contexts) is more central than the entropy in the output context alone. To allow for these and other possibilities in formulating theories of the cost function, a less committed variant would say that *C*(λ_*i*_, *c*) is a monotonically increasing function of S(*m*_*i*_) and H(*c*_*i*_).

## 4. Concluding Remarks

We have in (32) a function that assigns a cost to any given interpretation to an ambiguous sentence uttered in a context *c*. So far, I have said nothing about the disambiguation mechanism. I assume here that disambiguation decisions are made by finding optimal solutions to a coordination problem between speaker and hearer [see (6)]. In general, such decisions will involve assigning utilities to the space of output contexts, where coordination gets more utility than non-coordination and where the utilities might take the costs in (32) into account. There will also be a probability distribution over the space of output contexts (the probability that the speaker intends for each candidate to be the output context), and this will be partly determined by assumptions about the speaker's epistemic state. There will also be assumptions about what the QUD is, and these will determine (in conjunction with *exh* and the Maxim of Quantity) what the space of output contexts will be. In such a framework, the cost function puts a certain pressure to minimize costs (by the utility function), but the costs will be just one factor in the set of considerations that help a listener disambiguate. I should like to emphasize, however, that probabilities in this architecture only enter into disambiguation considerations, and hence the approach developed here is quite different than systems that allow probabilities to enter into the strengthening mechanism itself (e.g., Franke, 2011; Potts et al., 2015; Bergen et al., 2016). In the terminology of Fox and Katzir (2019), I assume that *exh* does not take a probability distribution as an argument, although the function that solves the decision problem in (6) does.

The cost function in (32) aims to make sense of CUPID, the puzzle of why and how exhaustification can be treated with uniformity in the competence system but with diversity in the performance system. I have argued that this can be made sense of by assuming that exhaustification itself is not the source of cost. Instead, I assume that costs are calculated by systems that ignore the computations internal to the language faculty. The cost calculation looks at the proposition denoted by each candidate analysis of the sentence, as well as the way this proposition would affect the information in the context, and assigns a cost to each using domain-general considerations. Like other models proposed from the early days of generative grammar (e.g., Miller and Chomsky, 1963) up to more modern treatments (e.g., Levy, 2013), my proposal here identifies a role for the complexity of the sentence itself as well as for information-theoretic reasoning about uncertainty resolution. However, the only aspect of the sentence that is relevant for our purposes is its meaning, with no regard for or access to its computational history.

The commitment to domain-general principles pursued here means that I have not considered language-dependent characterizations of scalar diversity in processing. For example, acquisition studies have argued that children differ from adults in one important way: they do not make lexical substitutions in generating *ALT* (e.g., Barner and Bachrach, 2010; Barner et al., 2011; Singh et al., 2016b; Tieu et al., 2017). One might pursue the idea that lexical substitutions, even when they emerge in the adult state, are the source of processing costs (see Chemla and Bott, 2014 and van Tiel and Schaeken, 2017 for steps in this direction). Note that free-choice inferences do not *require* lexical substitutions (the constituents are enough of a substitution source), and numerals cannot in general require lexical substitutions because the set of alternatives is infinite and hence must be generated by the successor function (see also section 1.4). This perspective would need to make sense of why lexical substitution does not seem to be hard with *only* (Marty and Chemla, 2013), and in any event working this all out raises non-trivial challenges that would take us too far afield to discuss here (Chemla and Singh, 2016). I hope to return to a fuller comparison in future work.

We have considered the idea that *exh* has several functions: it typically strengthens meanings, but it also often complicates meanings and gets us to better and better answers without having to verbalize them outright. Consider for example assertion of a disjunction *P*_*k*_ = *p*_1_ ∨ *p*_2_ ∨ …*p*_*k*_ in a world with no *exh* and in which the Maxim of Quantity governs communication. In such a world, you only eliminate one cell of the 2^*k*^ cells of the partition, and you thus generate lots of ignorance inferences (Fox, 2007). But suppose that the speaker in this world knows that exactly one of the *p*_*i*_ is true but doesn't know which. They would then have to produce a complex utterance to convey this thought: (*p*_1_ ∧ ¬*p*_2_ ∧ … ∧ ¬*p*_*k*_) ∨ (¬*p*_1_ ∧ *p*_2_ ∧ ¬*p*_3_ ∧ …¬*p*_*k*_) ∨ …(¬*p*_1_ ∧ … ∧ ¬*p*_*k*−1_ ∧ *p*_*k*_). This is a *k*^2^ mouthful. If a super-engineer were kind enough to give the speaker and hearer access to *exh*, they could communicate this complex piece of information by uttering *P*_*k*_ and hoping the listener would realize they should parse the sentence with *exh*. Presumably, the joint cost of *exh* and *P*_*k*_, together with the risk of error (given the new ambiguity), is a better way to communicate a good and complex answer than having to utter it outright.

## Data Availability Statement

All datasets generated for this study are included in the article/supplementary material.

## Author Contributions

The author confirms being the sole contributor of this work and has approved it for publication.

### Conflict of Interest

The author declares that the research was conducted in the absence of any commercial or financial relationships that could be construed as a potential conflict of interest.

## Footnotes

^1^Some proposals allow you to conjoin the basic meaning with unnegated alternatives (e.g., Chemla, 2009a; Bar-Lev and Fox, 2017). The differences between these theories will not concern us here (though see Note 24). What is important for current purposes is that strengthening occurs by conjoining a sentence with some other propositions derived from a restricted set of alternatives.

^2^It is known that *exh* has a restricted distribution (e.g., Singh, 2008a,b; Chierchia et al., 2012; Gajewski and Sharvit, 2012; Fox and Spector, 2018; Enguehard and Chemla, 2019). A more accurate characterization, then, is that *exh* can apply to any sentence in which it is licensed. All the examples we consider in this paper are ones in which *exh* is licensed.

^3^There are other possibilities here depending on what is assumed about the underlying parse. For example, if the *or* in the LF of (2) disjoins NPs instead of sentences, we would replace the noun phrase *cake or ice-cream* by each disjunct. The end result is the same in this case.

^4^In such cases, *ALT*(*p* ∨ *q*) = {*p, q*}. Let *S*_0_ be *p* ∨ *q* and let *A*_1_ be {*p, q*}. The first application of *exh* on *S*_0_ is vacuous because neither *p* nor *q* is innocently excludable: *exh*(*A*_1_, *S*_0_) is equivalent to *p* ∨ *q*. Let *S*_1_ be the sentence *exh*(*A*_1_, *S*_0_), and consider the exhaustification of *S*_1_: *exh*(*ALT*(*S*_1_), *S*_1_). The alternatives here are {*exh*(*A*_1_, *p*), *exh*(*A*_1_, *q*)} = {*p* ∧ ¬*q, q* ∧ ¬*p*}. Both are innocently excludable, and hence *exh*(*ALT*(*S*_1_), *S*_1_) is equivalent to *p* ∧ *q*.

^5^Some people have argued that sentences are always parsed with *exh* (e.g., Magri, 2009, 2011; Crnič et al., 2015). The observation that sentences aren't always strengthened is accounted for by appealing to contextual domain restriction in the alternatives. Everything we say here could be suitably translated into such a framework. For example, let *A* be *ALT*(∃) = {∀}, and let *B* be the result of contextual pruning of *ALT*(∃): *B* = ∅. Thus, instead of comparing ∃ and *exh*(*A*, ∃) we would compare *exh*(*A*, ∃) and *exh*(*B*, ∃). Because our concern is only with the *meanings* of candidates, and not with their forms/computational histories, the proposal here could readily accommodate the assumption that *exh* is mandatory (along with competing ideas about strengthening). I will continue to assume here that *exh* is part of the inventory of logical operators and that its application is optional.

^6^Emmanuel Chemla (p.c.) notes that even under a parallel model it is conceivable that the parser could sometimes decide to stop at the smaller < *S*, [[*S*]] >, and this could account for the average cost difference. Like with the serial model, much depends on the “order” in which *exh* is applied. For example, one could design a parallel parser that creates parses top-down such that *exh*(*S*) and *S* are always in the set of possibilities together, among other choice points.

^7^Note that *ALT*((4)) = {◇*p*, ◇*q*, ◇(*p* ∧ *q*)} is not closed under conjunction. Hence, we expect recursive exhaustification to yield the conjunctive free-choice scalar implicature ◇*p* ∧ ◇*q* (the reader can use note 4 to work this out).

^8^Podlesny (2015) argues that similar facts in American Sign Language follow the same pattern (though cf. Davidson, 2013). The pattern in question here is that disjunctive sentences can receive a free-choice (conjunctive) strengthened meaning when their alternatives are not closed under conjunction (see Fox, 2007; Chemla, 2009a; Franke, 2011; Singh et al., 2016b) and note 4.

^9^More generally, one is interested in functions that map {0, 1}^*D*^ to {0, 1}^*D*′^. We will not pursue this more general framework (see e.g., Savage, 1976).

^10^Of course, different primitives will give rise to different complexity measures. For example, exclusive disjunction requires at least four literals in a language with just ∧ , ∨ , ¬ [see e.g., (9-d) and (9-e)]. Note that negation is not ‘counted' in the measure—the motivation for this is that *p* and ¬*p* divide logical space in the exact same way (Feldman, 2000). If exclusive disjunction were a primitive, ⊕ say, then you could get away with just two literals. Different complexity measures could also be considered. For example, the current measure does not count operators; some other measures would, such as ones that associate Boolean functions with complexity measures relating to the size or depth of circuits that compute them (see e.g., Sipser, 1997). For current purposes, I will assume that the concept learning literature (in particular Feldman, 2000) provides sufficient motivation for assuming that the set of primitives assumed here is telling, as is the assumed complexity measure. Note also that morphologically simplex operators in natural language appear to be restricted to just these primitives (Katzir and Singh, 2013). For relevant discussion, see also Piantadosi et al. (2016), Buccola et al. (2018), and note 16.

^11^It is sometimes argued that a large number of alternatives needs to be accessed during exhaustification, and that this could lead to computational costs (e.g., Mascarenhas, 2014; Spector, 2016). Note that the possibility of embedded exhaustification provides a significant reduction in the number of alternatives that need to be considered. Here we have one *k*-membered set, which gets used *k* times in exactly the same way each time. A global exhaustification using innocent exclusion could derive the same results by closing *C* under conjunction and ignoring closure under disjunction (see results in Spector, 2016). In fact, combinatorial explosion is at its worst when all we can do is blindly search through the entire space. This is not necessarily so with *ALT*, because there is sufficient structure within *ALT* that a sophisticated reasoner could exploit. For example, when finding maximal consistent exclusions (Fox, 2007), as soon as you decide that *p* ∧ *q* is excludable, say, you can automatically conclude that any alternative *r* in which *p* ∧ *q* is a subformula is also excludable (because *r* entails *p* ∧ *q*). Thus, algorithms for solving innocent exclusion might be able to avoid “perebor” (brute-force exhaustive search, no pun intended; cf. Trakhtenbrot, 1984). I will thus continue to assume that only the output of the language faculty is relevant to cost considerations. If it turns out that the number of alternatives is relevant, our cost formulation will have to change.

^12^An interesting question is whether *exh* is always monotonic in semantic complexity. There is no logical necessity to this: a reviewer points out that *exh*(*p* ⊕ *q*, {*q*}) means *p* ∧ ¬*q*, which is simpler than *p* ⊕ *q*. The question of interest here is an empirical one: are there any cases of natural language sentences *S* such that *exh*(*S, ALT*(*S*)) has lower Boolean Complexity than *S*?

^13^Some quantifiers like *most* require push-down automata. There are close parallels between first-order definability and the Chomsky hierarchy. See van Benthem (1986).

^14^More generally, a finite state machine is characterized by: (i) a finite set of states Q; (ii) an alphabet Σ; (iii) a transition function δ : Q × Σ → *Q* describing how the machine moves; (iv) a start state *q*_1_ ∈ Q; and (v) a set of accept states F ⊆ *Q*. The machine in (12) has the following description: (i) Q = {*q*_1_, *q*_2_}; (ii) Σ = {0, 1}, (iii) δ maps (*q*_1_, 0) to *q*_1_, (*q*_1_, 1) to *q*_2_, (*q*_2_, 0) to *q*_2_, and (*q*_2_, 1) to *q*_2_; (iv) *q*_1_ is the start state, and (v) F = {*q*_2_} is the (singleton) set of accept states. See any introductory text on formal language theory or the theory of computation for more detailed discussion of the properties of such machines (e.g., Sipser, 1997).

^15^A reviewer points out that (13) also accepts the empty string (the empty string is always accepted by machines for which the start state is an accept state). I omit mention of the empty string in the main text to avoid clutter and to simplify exposition. The reviewer notes that the machine here does not take existential import into account; without existential import, *all* would not entail *some*. The reviewer notes that a three-state machine would capture existential import. I believe we can sidestep the question of existential import because entailment is not needed for our purposes. As formulated in Fox (2007), *exh* negates not only stronger alternatives, but also those that are merely non-weaker. Either way, this will not affect our main point about the costs of strengthening *some* (though see note 16). I hope this makes it okay to ignore the empty string and its complications in the main text.

^16^Buccola et al. (2018) conclude from their results that ∀, unlike ∃^+^, is a plausible candidate for being a primitive in the language of thought. I will not enter into full discussion here, but it might be interesting to explore the connection between semantic complexity and logical primitives. If simplex lexical items are restricted to logical/conceptual primitives, then semantic complexity does not uniquely identify the primitives, given the existence of semantically simple but unlexicalized elements like *nand* (= ¬∧), *nall* (= ¬∀), and others (e.g., Horn, 1972; Katzir and Singh, 2013). Furthermore, learnability arguments suggest that a logical primitives approach (as in Keenan and Stavi, 1986) can be dissociated from semantic complexity (see Katzir and Peled, 2018).

^17^More generally, machines accepting “at least *n*” require *n* + 1 states and those accepting “exactly *n*” require *n* + 2 states. Roni Katzir (p.c.) points out that the machine for “exactly *n*” could be simplified if we remove arrows leading to “sink states,” i.e., non-accepting states like *q*_4_ in (18) from which there is no escape. If we were to do this, the machines in (17) and (18) would have the same complexity. The empirical problem at hand would remain even if we adopted this way of counting: we need to account for the observation that the exactly-reading of numerals appears to not only be free (relative to its basic meaning counterpart), but in fact less costly than it (Marty et al., 2013). I will thus continue to assume that sink states are included in the complexity measure, and hope that we could restate things if it turns out that removing them would be preferred.

^18^Nor does the alleged primitiveness of ∀, assuming that ∃ is also primitive (cf. Note 16).

^19^It is also easy to tell whether the uttered sentence's presupposition is satisfied or is otherwise innocuous (just compare the presupposition with the information in the common ground). When it is not, there are detectable and immediate costs for accommodation (e.g., Singh et al., 2016a).

^20^See also Katzir and Singh, 2015 for a related but somewhat different notion of the “goodness” of answers, together with suggestions about the goodness of questions as well.

^21^There might be more propositional variables under consideration, but this doesn't affect anything we have to say here.

^22^Fox (2007) notes that the pure Maxim of Quantity leads only to ignorance inferences about all relevant propositions whose truth-values are not settled by the speaker's utterance. This in turn follows from considerations of relevance (the so-called ‘symmetry problem'; cf. von Fintel and Heim, 1999). In Fox's (2007) system, exhaustivity is a mechanism that helps conversational participants take sentences that are at best partial answers and convert them into better partial answers or into complete answers where possible (see especially Fox, 2018 for extensive discussion with consequences for the semantics and pragmatics of questions more generally).

^23^A reviewer raises the question of how we can identify the QUD of an utterance. For example, consider a context in which the goalkeeper Sue must not let in more than 2 goals to keep her position as starting keeper. A asks: *Did Sandy keep her position?* B responds: *No, she let in three goals*. In a sense, the strengthened meaning of B's response gives strictly more information than is required to answer A's question, whereas the basic meaning itself gives exactly the right amount. The reviewer wonders whether the QUD for *she let in three goals* might nevertheless be a *how many* question. There is certainly room for flexibility of QUDs, and numerals might strongly be associated with *how many* questions. At the same time, we have not said anything about how to incorporate an overly strong answer in our measure of “distance from a complete answer.” I leave this as a challenge for now.

^24^Bar-Lev and Fox (2017) propose that “innocent inclusion”—a new method for computing free-choice—is obligatory and hence cost-free while “innocent exclusion” (Fox, 2007)—used for more standard scalar implicatures (like ∃^+^)—has a cost due to context-sensitive optionality. The case of numerals suggests that complete answerhood is the more fundamental notion. Of course, this does not speak at all to the motivation for introducing innocent inclusion in the first place (the need for a global mechanism to compute universal free choice—Chemla, 2009b).

^25^Shannon (1948) posited some basic axioms that any measure of relevant uncertainty should follow, and proved that (29) is the unique measure satisfying these axioms. Throughout this paper, we will assume that our logarithms are binary (*log*_2_*n* is that number *k* such that 2^*k*^ = *n*).

^26^To reduce clutter, we omit the multiplicative constant that is sometimes presented in the derivation of entropy.

^27^This assumption might turn out to be problematic. It is conceivable that probabilities decrease with the number of true alternatives. For example, an *a priori* assumption that predicate extensions are as small as possible might provide a rationale for theories of “minimal worlds/models” theories of exhaustivity (e.g., van Benthem, 1989; van Rooij and Schulz, 2004; Spector, 2005, 2006, 2016; Schulz and van Rooij, 2006). Given the symmetry problem (von Fintel and Heim, 1999; see also Fox (2007); Katzir (2007)), the Maxim of Quantity cannot motivate the minimal worlds/models assumption. For example, suppose we learn from a speaker that *R*(*a*) and we are in a context in which it is relevant whether *b* satisfies *R*. A speaker obeying the Maxim of Quantity could only be taken to be ignorant about whether *R*(*b*). However, if *R*(*b*) is *a priori* less likely than ¬*R*(*b*), this might make it rational for the listener to conclude that *R*(*b*) is false. More generally, it is plausible to assume that for an arbitrary predicate *P* and arbitrary individual *c*, *P*(*c*) is less likely to be true than false. This assumption may relate to the “size principle” proposed in concept learning (e.g., Tenenbaum, 1999), and may also underlie our ability—granted by *exh*—to state only the positive instances of a predicate (these being the least likely, and hence worth the cost of expression). See also Bar-Hillel and Carnap (1952) on (a-)symmetries between a predicate and its negation. I hope to return to this set of ideas in future work.

^28^More generally, the *noiseless coding theorem* states that the minimal average code length for encoding outcomes in Q is very close to the entropy of Q.

^29^Roni Katzir (p.c.) notes that the picture here is quite analogous to *Minimum Description Length* (MDL) approaches to learning (Rissanen, 1978). Such approaches compare competing hypotheses for a given set of data by minimizing the sum of (i) the cost to encode the hypothesis, and (ii) the cost to encode the data given the hypothesis. Semantic complexity straightforwardly relates to (i), but it is unclear (to me) how to relate entropy to (ii). For example, in MDL learning we compare hypotheses that can make sense of the data. In our disambiguation scenario, we have different form-meaning pairs that can be associated with the observable data (the sentence *S*), but what the entropy measure is concerned with is how these different analyses affect the context, and different analyses will (in general) lead to different contexts. I hope to return to the comparison with MDL in future work.
